# Supplementary material for: Malaria prevention knowledge, attitudes, and practices (KAP) among adolescents living in an area of persistent transmission in Senegal: Results from a cross-sectional study
Source: PLoS One. 2022 Dec 1;17(12):e0274656. doi: 10.1371/journal.pone.0274656 (PMC9714833; doi:10.1371/journal.pone.0274656)
Supplement: S1 File — (DOCX) [file pone.0274656.s001.docx]

**English version questionnaire**

Date of survey: ___/___/_______/

Initials of investigator: ________________________________________

| 1. **PARTICIPANT IDENTIFICATION** | | | | | | |
| --- | --- | --- | --- | --- | --- | --- |
|  | Health post | 1. Diakhaling 2. Khossanto 3. Mamakhono 4. Sambrambougou | | | | |
|  | Village | _____________________________ | | | | |
|  | Participant ID | I____I____I____I____I____I____I | | | | |
|  | Ethnicity | 1. Sarakole 2. Wolof 3. Pular 4. Bassari 5. Other, specify __________________ | | | | |
| 5. | Age | I____I____I years | | | | |
| 6. | Date of birth | I____I____I____I____I____I____I____\|  (indicate date of birth if age is not available) | | | | |
| 7. | Gender | 1. \|___\| Male 2. \|___\|. Female | | | | |
| 8. | Occupation | 1. Student 2. Shepherd 3. Gold digger 4. Teacher 5. Farmer 6. Seller 7. Driver 8. Other, specify _______________________ | | | | |
| 9.  9. | Level of education | 1. Koranic School 2. Primary 3. Secondary 4. University 5. None 6. Other, specify _______________________ | | | | |
| 1. **Identification of the participant's head of household (IF THE PARTICIPANT is not the head of household)** | | | | | | |
|  | Head of household initials | **______________________** | | | | |
|  | Level of education | 1. Koranic school 2. Primary level 3. Secondary level 4. University level 5. None 6. Other, specify _______________________ | | | | |
|  | Occupation | 1. Shepherd 2. Gold digger 3. Teacher 4. Farmer 5. Seller 6. Driver 7. Other, specify __________________________________ | | | | |
|  | Gender | 1. \|___\| Male 2. \|___\|. Female | | | | |
| 1. **Household characteristics** | | | | | | |
|  | Number of people in the household | \|___\|___\| | | | | |
|  | Type of roof | 1. Metal sheet 2. Cement/Beton 3. Straw/stubble 4. Cardboard 5. Boards 6. Other, specify __________________ | | | | |
|  | Type of wall | 1. Cement 2. Rammed earth/mud 3. Wood/boards 4. Bamboo 5. Other, specify _________________________ | | | | |
|  | Type of floor | 1. Cement 2. Tiles 3. Sand 4. Wooden boards 5. Other, specify _______________________ | | | | |
|  | Water source | 1. Tap 2. Well in the house 3. Public well 4. Tanker truck 5. Rainwater 6. Other, specify _______________________ | | | | |
|  | Type of toilet | 1. Personal toilet 2. Common toilet 3. Personal latrine 4. Common latrine 5. No toilet 6. Other, specify ___________________________ | | | | |
|  | Type of cooking fuel | 1. Firewood 2. Gas 3. Electricity 4. petroleum 5. Coal 6. Other, specify _________________________ | | | | |
| 1. **Possession of property** | | | | | | |
|  | Radio | \|____\| Yes/No | | | | |
|  | Television | \|____\| Yes/No | | | | |
|  | Bike | \|____\| Yes/No | | | | |
|  | Scooter | \|____\| Yes/No | | | | |
|  | Car | \|____\| Yes/No | | | | |
|  | Refrigerator | \|____\| Yes/No | | | | |
|  | Fan | \|____\| Yes/No | | | | |
|  | Mobile phone | \|____\| Yes/No | | | | |
|  | Carts | \|____\| Yes/No | | | | |
|  | Cattle | \|____\| Yes/No | | | | |
| 1. **Malaria prevention** | | | | | | |
|  | Do you have bed nets in the household? | 1. Yes 2. No | | | | |
|  | How many (skip if Q1 =No) | \|____\| | | | | |
|  | Do you sleep under a bed net? | 1. Yes 2. No | | | | |
|  | Why, don’t you sleep under a bed net?  (if Q3=No) | 1. Very hot 2. Do not like the smell 3. I feel "trapped in the bed net." 4. No malaria currently 5. No mosquitoes 6. 6. Bed net is too old and torn 7. Other, specify _____________________ | | | | |
|  | In which season, do you sleep under  a bed net? (skip if Q3=No) | 1. Rainy season 2. Dry season 3. All seasons 4. Don’t know | | | | |
|  | How often do you sleep under a bed net?  (skip if Q3=No) | 1. Every night 2. 3 to 6 times a week 3. less than 3 times (0, 1, 2) per week | | | | |
|  | Did you sleep under a bed net last night? | 1. Yes 2. No | | | | |
|  | Why, didn’t you sleep under a bed net  last night? (if Q7=No) | 1. Very hot 2. Do not like the smell 3. I feel "trapped in the bed net." 4. No malaria currently 5. No mosquitoes 6. Bed net is too old and torn 7. Other, specify _____________________ | | | | |
|  | Do you use other means of prevention? | 1. Yes 2. No | | | | |
|  | Indicate the other means you use (if Q9=Yes) | 1. Smoke coil 2. insecticide (e.g. yotox) 3. Tall grass cleaning 4. Sewage disposal 5. Wearing long clothes 6. Other, specify ___________________ | | | | |
| 1. **Knowledge** | | | | | | |
|  | Have you ever heard about malaria? | 1. Yes 2. No | | | | |
|  | By which source have you heard about malaria? (if Q1=Yes) | 1. Radio 2. TV 3. School 4. ASC 5. ICP 6. Other, specify _____________________ | | | | |
|  | How is malaria transmitted (causes)?  (if Q1=Yes) | 1. Sleeping with a sick person 2. Mosquito bite 3. Insect bite 4. Contaminated food 5. Lack of personal hygiene 6. Oil consumption 7. Sun exposure 8. Other, specify _____________________ 9. Don’t know | | | | |
|  | What are the symptoms of malaria?  (if Q1=Yes) | 1. Headache 2. Abdominal pain 3. Chills 4. Fatigue 5. Lack of appetite 6. Abdominal pain 7. Vomiting 8. Other, specify _____________________ 9. Don’t know | | | | |
|  | How can malaria be prevented?  (methods of malaria prevention) (if Q1=Yes) | 1. Mosquito net 2. Insecticide (e.g. yotox) 3. Smoke coil 4. Anti-malarial 5. Weeding 6. Wearing long clothes 7. Sewage disposal 8. Fan 9. Other, specify _____________________ 10. Don’t know | | | | |
| 1. **Attitudes** | | | | | | |
|  |  | Totally agree | I agree | Neutral | Disagree | Strongly disagree |
|  | Everyone can get malaria |  |  |  |  |  |
|  | Malaria is deadly |  |  |  |  |  |
|  | Malaria can be cured without medical treatment |  |  |  |  |  |
|  | Malaria is preventable |  |  |  |  |  |
|  | It is important to confirm the diagnosis of malaria at the health post before treatment |  |  |  |  |  |
|  | It is necessary to finish a treatment against malaria |  |  |  |  |  |
| **E. Treatment practices (care seeking)** | | | | | | |
|  | Have you had malaria recently? | 1. Yes 2. No | | | | |
|  | How did you know that you had malaria?  (skip if Q1=No) | 1. Diagnosis at the health post/center/DSDOM 2. Drug received 3. Symptoms 4. Other, specify ____________________ | | | | |
|  | Did you seek care for this episode?  (skip if Q1=No) | 1. Yes 2. No | | | | |
|  | Why didn’t you seek care?  (skip if Q3=Yes) | 1. Lack of money 2. Lack of time 3. The distance (too far) 4. It was not necessary 5. I don't know where to go 6. Other, specify ________________ | | | | |
|  | Where did you seek care? (if Q3=Yes) | 1. Health post 2. Health center 3. Health hut/DSDOM 4. Traditional practitioners 5. Modern/traditional self-medication 6. Pharmacy 7. Other, specify ________________ | | | | |
|  | How long after the symptoms did you seek care? (if Q3=Yes) | 1. On the same day 2. One day 3. Two days after 4. Other, specify_________________________ 5. Don’t Know | | | | |
|  | Proximity to a functional health structure  (How long does it take to get to the health post?) | 1. Less than one hour (< 60 min) 2. More than one hour (> 60 min) | | | | |
