# Supplementary material for: Malaria prevention knowledge, attitudes, and practices (KAP) among adolescents living in an area of persistent transmission in Senegal: Results from a cross-sectional study
Source: PLoS One. 2022 Dec 1;17(12):e0274656. doi: 10.1371/journal.pone.0274656 (PMC9714833; doi:10.1371/journal.pone.0274656)
Supplement: S2 File — (DOCX) [file pone.0274656.s002.docx]

**French version questionnaire**

Date de l’enquête: ___/___/_______/

Nom de l’enquêteur: ________________________________________

|  | 1. **IDENTIFICATION DU PARTICIPANT** | | | | | |
| --- | --- | --- | --- | --- | --- | --- |
|  | Poste de santé | 1. Diakhaling 2. Khossanto 3. Mamakhono 4. Sambrambougou | | | | |
|  | Village |  | | | | |
|  | Code du participant | I____I____I____I____I____I____I____I____\| (copier le numéro sur l’étiquette) | | | | |
|  | Ethnie | 1. Sarakole 2. Wolof 3. Pular 4. Bassari 5. Autres, spécifiez | | | | |
|  | Age | I____I____I années | | | | |
|  | Date de naissance | I____I____I____I____I____I____I____I____\| indiquer la date de naissance si l’âge n’est pas disponible) | | | | |
| - - 1. 6. | Sexe | 1. \|___\| Masculin 2. \|___\|. Féminin | | | | |
|  | Occupation | 1. Élève 2. Berger 3. Orpailleur 4. Enseignant 5. Cultivateur 6. Vendeur/Commerçant 7. Chauffeur de Taxi 8. Autres, spécifiez | | | | |
|  | Niveau d’éducation | 1. École coranique 2. Primaire 3. Secondaire 4. Universitaire 5. Aucune 6. Autre, spécifiez | | | | |
| 1. **Identification du chef de ménage du participant (SI LE PARTICIPANT n’est pas le chef de ménage)** | | | | | | |
|  | Initiales du chef de ménage |  | | | | |
|  | Niveau d’éducation | 1. École coranique 2. Niveau primaire 3. Niveau secondaire 4. Niveau universitaire 5. Aucune 6. Autre, spécifiez_______________________ | | | | |
|  | Occupation | 1. Berger 2. Orpailleur 3. Enseignant 4. Cultivateur 5. Vendeur/Commerçant 6. Chauffeur de Taxi 7. Autres, spécifiez __________________________________________ | | | | |
|  | Sexe | 1. \|___\| Masculin 2. \|___\|. Féminin | | | | |
| 1. **Caractéristiques du ménage** | | | | | | |
|  | Nombre de personnes dans le ménage | \|___\|___\| | | | | |
|  | Type de toit | 1. Tôle 2. Ciment/Béton 3. Paille/chaume 4. Carton 5. Planches 6. Autres, spécifiez | | | | |
|  | Type de mur | 1. Ciment 2. Terre battue/boue 3. Bois/planches 4. Bambou 5. Autres : _________________________ | | | | |
|  | Type de sol | 1. Ciment 2. Carreaux 3. Sable 4. Planches de bois 5. Autres : _______________________ | | | | |
|  | Source d’eau | 1. Robinet, 2. Puits dans la maison, 3. Puits public 4. Camion-citerne 5. Eau de pluie 6. Autres: _______________________ | | | | |
|  | Type de toilette | 1. Toilette personnelle 2. Toilette commune 3. Latrine personnelle 4. Latrine commune 5. Absence de toilette 6. Autre ___________________________ | | | | |
|  | Type de combustible de cuisson | 1. Bois de chauffage 2. Gaz 3. Électricité 4. Pétrole 5. Charbon 6. Autres : _________________________ | | | | |
|  | 1. **Possession de biens** | | | | | |
|  | Radio | \|____\| Oui/non | | | | |
|  | Télévision | \|____\| Oui/non | | | | |
|  | Vélo | \|____\| Oui/non | | | | |
|  | Scooter | \|____\| Oui/non | | | | |
|  | Voiture | \|____\| Oui/non | | | | |
|  | Réfrigérateur | \|____\| Oui/non | | | | |
|  | Ventilateur | \|____\| Oui/non | | | | |
|  | Téléphone portable | \|____\| Oui/non | | | | |
|  | Charrettes | \|____\| Oui/non | | | | |
|  | Bétails | \|____\| Oui/non | | | | |
|  | 1. **Prévention du paludisme** | | | | | |
|  | Possédez-vous des moustiquaires dans le ménage? | 1. Oui 2. Non | | | | |
|  | Combien (sauter si Q1=Non) | \|  \| \| --- \| | | | | |
|  | Dormez-vous sous moustiquaire? | 1. Oui 2. Non | | | | |
|  | Pourquoi ne dormez pas vous sous la moustiquaire?(si Q3=Non) | 1. Oui 2. Non | | | | |
|  | A quelle saison, dormez-vous sous moustiquaire (sauter si Q3=Non) | 1. Saison pluvieuse 2. Saison sèche 3. Toutes les saisons 4. NSP | | | | |
|  | A quelle fréquence, dormez-vous sous moustiquaire? (sauter si Q3=Non) | 1. Toutes les nuits 2. 3 à 6 fois par semaine 3. moins de 3 fois (0, 1, 2 fois) par semaine | | | | |
|  | Avez- vous dormi sous moustiquaire la nuit dernière? | 1. Oui 2. Non | | | | |
|  | Pourquoi? (si Q7=Non) | 1. Très chaud 2. N’aime pas l’odeur 3. Je me sens «enfermé » 4. Pas de paludisme actuellement 5. Pas de moustiques 6. Moustiquaire trop veille et déchirée 7. Autre _____________________ | | | | |
|  | Utilisez-vous d’autres moyens de prévention? | 1. Oui 2. Non | | | | |
|  | Indiquez les autres moyens que vous utilisez (Si Q9=Oui) | 1. Serpentin fumigène 2. Insecticide (ex. yotox) 3. Nettoyage des hautes herbes 4. Évacuation des eaux usées 5. Port d'habits longs 6. Autres, spécifiez | | | | |
| 1. **Connaissances** | | | | | | |
|  | Avez-vous déjà entendu parler du paludisme? | 1. Oui 2. Non | | | | |
|  | Par quelle source (si Q1=Oui)? | 1, Radio  2, TV  3, École  4, ASC  5, ICP  6, Autre, spécifiez | | | | |
|  | Comment le paludisme est-il transmis (causes)?(si Q1=Oui) | 1. Dormir avec une personne malade 2. Piqûre de moustiques 3. Piqûre d'insecte \| 4. Aliments contaminés 5. Manque d'hygiène personnelle 6. Consommation d'huile 7. Exposition au soleil 8. Autres 9. NSP | | | | |
|  | Quels sont les symptômes du paludisme? (si Q1=Oui) | 1. Maux de tête 2. Douleurs abdominales 3. Frissons 4. Fatigue 5. Manque d'appétit 6. Douleurs abdominales 7. Vomissement 8. Autres, spécifiez 9. NSP | | | | |
|  | Comment peut-on prévenir le paludisme? (méthodes de prevention du paludisme) (si Q1=Oui) | 1. Moustiquaire 2. Insecticide (ex. yotox) 3. Serpentin fumigène 4. Antipaludique 5. Désherbage 6. Port d'habits longs 7. Évacuation des eaux usées 8. Ventilateur 9. Autres 10. NSP | | | | |
| 1. **Attitudes** | | | | | | |
|  |  | Tout à fait d’accord | D'accord | Neutre | Pas d'accord | Pas du tout d'accord |
|  | Tout le monde^[[1]](#footnote-1)^ peut contracter le paludisme |  |  |  |  |  |
|  | Le paludisme est mortel |  |  |  |  |  |
|  | Le paludisme peut se guérir sans traitement médical |  |  |  |  |  |
|  | On peut prévenir le paludisme |  |  |  |  |  |
|  | Il est important de confirmer le diagnostic du paludisme au poste de santé avant le traitement |  |  |  |  |  |
|  | Il est nécessaire de finir un traitement contre le paludisme |  |  |  |  |  |
| 1. **Pratiques en matière de traitement (recherche de soins)** | | | | | | |
|  | Avez-vous eu le paludisme récemment? | 1. Oui 2. Non | | | | |
|  | Comment avez-vous su? (sauter si Q1=Non) | 1, Diagnostic au poste de santé/case de santé/ DSDOM  2, Médicament reçu  3, Symptômes  4, Autres, spécifiez | | | | |
|  | Êtes allés aux soins? (sauter si Q1=Non) | 1. Oui 2. Non | | | | |
|  | Pourquoi vous n’êtes pas allés aux soins? (sauter si Q3=Oui) | 1. Manque d'argent 2. Manque de temps 3. La distance (trop loin) 4. Ce n'était pas nécessaire 5. Je ne sais pas où aller 6. Autres, spécifiez | | | | |
| 5. | Où êtes-vous rendus pour se soigner? (si Q3=Oui) | 1. Au poste de santé 2. Au centre de santé 3. À la case de santé/Chez le DSDOM 4. Chez les tradipraticiens 5. Auto-médication moderne/traditionnelle 6. Pharmacie 7. Autres, spécifiez | | | | |
| 6. | Combien de temps, après les symptômes êtes-vous allés chercher les soins ? (si Q3=Oui) | 1. Dans la journée même 2. Un jour 3. Deux jours après 4. Autres: _________________________ 5. NSP | | | | |
|  | Proximité d’une structure sanitaire fonctionnelle (Combien de temps faut-il pour vous rendre au poste de santé ? | 1. Moins d’une heure (< 60 min) 2. Plus d’une heure (> 60 min) | | | | |

1. Tout le monde inclut personnes agées, femmes enceintes, les enfants, etc..) [↑](#footnote-ref-1)
