# Supplementary material for: Malaria prevention knowledge, attitudes, and practices (KAP) among adolescents living in an area of persistent transmission in Senegal: Results from a cross-sectional study
Source: PLoS One. 2022 Dec 1;17(12):e0274656. doi: 10.1371/journal.pone.0274656 (PMC9714833; doi:10.1371/journal.pone.0274656)
Supplement: S3 File — (DOCX) [file pone.0274656.s003.docx]

**Ñininkaari laŋo**

Ñininkaroo waatoo: ___/___/_______/

Ñininkaarilaa: ________________________________________

|  | 1. **Maabeelaa la loŋo** | | | | | |
| --- | --- | --- | --- | --- | --- | --- |
|  | Jaaraalidulaa | 1. Jaakaliŋ 2. kosanto 3. Mamakono 4. Sambranbugu | | | | |
|  | Saatee |  | | | | |
|  | Maabeelaa/mantaa | I____I____I____I____I____I____I (Ka a la suteeraŋ safee) | | | | |
|  | Sio | 1. Sooninke 2. Wolof 3. Pulaar 4. Oniyan 5. waraŋ doo, Isafee | | | | |
|  | Saanjoo | I____I____I Saŋo | | | | |
|  | Wulu saŋo | I____I____I____I____I____I____I____I____\| walu waatoo safee niŋ wulu saŋo maŋ loŋ | | | | |
|  | muluŋo | 1. \|___\| Keo 2. \|___\|. Musu | | | | |
|  | Keekuo | 1. karandiŋo 2. kanlarilaa 3. sanisinnaa 4. karandirilaa 5. senelaa, doobaa 6. waafirilaa/fiirilaa 7. kuluŋ borindilaa 8. waraŋ doo I safee | | | | |
|  | Karaŋ tenboo | 1. Moori karaŋo 2. Tubaabu karaŋo talaa 1 3. Tubaabu karaŋo talaa 2 4. Tubaabu karaŋo Iniwerisite 5. Hani dulaa 6. waraŋ doo I safee | | | | |
|  | 1. **Maabeelaa suutiyoo la suuteeraŋo (Niŋ maabeelaa maŋ ke suutiyoo ti)** | | | | | |
|  | Suutiyoo suuteeraŋolu |  | | | | |
|  | Karaŋ tenboo | 1. Moori karaŋo 2. Tubaabu karaŋo talaa 1 3. Tubaabu karaŋo talaa 2 4. Tubaabu karaŋo Iniwerisite 5. Hani dulaa 6. waraŋ doo I safee _________________ | | | | |
|  | Kekuo | 1. karandiŋo 2. sanisinnaa 3. karandirilaa 4. senelaa, doobaa   5. waafirilaa/fiirilaa  6. kuluŋ borindilaa  7. waraŋ doo I safe___________________ | | | | |
|  | muluŋo | 1. \|___\| Keo 2. \|___\|. musu | | | | |
|  | 1. **Sinkiroo keeñaa** | | | | | |
|  | Sinkiroo moo yaatee | \|___\|___\| | | | | |
|  | Kankaraqo siifaa | 1. korikeetoo 2. Simenti buŋo 3. Nanlaq buŋo 4. Kayiti bantandiŋo 5. yiroo 6. waraŋ doo I safee | | | | |
|  | Padiiroo siifaa | 1. simentoo 2. potopoto buŋo/ ñukaa buŋo 3. yiroo 4. boŋo 5. waraŋ doo : _____________________ | | | | |
|  | Buŋ teeroo | 1. Simento 2. karoo 3. bankoo 4. yiroo 5. waraŋ doo : _____________________ | | | | |
|  | Jii solodulaa | 1. Jii bunluŋo, 2. Suukono koloŋo, 3. Saateebee koloŋo 4. Jii dunboo woloo 5. sanjio 6. waraŋ doo: _____________________ | | | | |
|  | Jonkoŋo siifaa | 1. Fansuŋ jonkoŋo 2. Moo bee jonkoŋo 3. Fansuŋ kabinee ( kamoo) 4. Moo bee kabinee (kamoo) 5. Kamoo tee daamiŋ 6. Waraŋ doo ______________________ | | | | |
|  | Tabirilaŋo | 1. Loo tabirilaŋo 2. gaasoo 3. kuraŋo dimbaa 4. karisiinoo 5. kenboo 6. waraŋ doo : ______________________ | | | | |
|  | 1. **Jooraŋolu miŋ nul sototaa** | | | | | |
|  | rajoo | \|____\| Haa/Hani | | | | |
|  | telee | \|____\| Haa/Hani | | | | |
|  | weloo | \|____\| Haa/Hani | | | | |
|  | Moto simmaa | \|____\| Haa/Hani | | | | |
|  | Kuluŋ borilaa | \|____\| Haa/Hani | | | | |
|  | Jii sumaa jooraŋo | \|____\| Haa/Hani | | | | |
|  | Foñoo sumaa jooraŋo | \|____\| Haa/Hani | | | | |
|  | Kiliraŋo/ poritabuloo | \|____\| Haa/Hani | | | | |
|  | sareetoo | \|____\| Haa/Hani | | | | |
|  | beeyaŋolu | \|____\| Haa/Hani | | | | |
|  | 1. **Kirikiroo fankantoo ñaatoosio** | | | | | |
|  | Fo sankee be ali bulu le sikiroo too? | 1. haa 2. hani | | | | |
|  | Jelu loŋ (sawuŋ niŋ Jaabiroo muŋ=hani ti) | \|  \| \| --- \| | | | | |
|  | Fo ali ka siinoo sankee koto le? | 1. haa 2. hani | | | | |
|  | Munne yaa tiŋ ali buka siinoo sankee koto?( sawuŋ niŋ jaabiri 3=hani ti) | 1. haa 2. hani | | | | |
|  | Tuma jumaa le ali ka I laa sanke koto (sawuŋ niŋ j3=hani ti) | 1. samaa waatoo, 2. tili kandoo, 3. waaloolu bee 4. waraŋ doo i safee | | | | |
|  | Waati jumaa le ali ka I laa sankee koto? (sawuŋ niŋ j3=hani ti) | 1. Suutoolu bee 2. Siiña 3 foo siiña 6 lookuŋo too 3. Miŋ maŋ siiña 3 sii lookuŋ too ( siiña 0, 1, 2) | | | | |
|  | Fo niŋ suuta tanbilaa ali ye I laa sanke koto le ? | 1. haa 2. hani | | | | |
|  | Munne ye atinna ? (niŋ j7=hani ti) | 1. Kando warata 2. maŋ lafi a nooroo la 3. Akatiŋ «n’bee sitiriŋ/sorondine » 4. Kirikiroo te keeriŋ 5. Sankeelu maŋ soto 6. Sankeelu kotoota le i farata 7. Waraŋ doo _____________________ | | | | |
|  | Ali ka fankantoo ñaatoo sii le baŋ? | 1. haa 2. hani | | | | |
|  | Ali feero doo dii ali ka munnu ke (niŋ j9=haa ti) | 1. Suusuulaa bori ŋoribontinma 2. Suusulaa bori junjunlaa (ex. yotox) 3. Ñaama baaroo 4. Jii loo dulaalu sukki 5. Dondika yiruwaa duŋ 6. Waraŋ doo kalaŋo | | | | |
|  | 1. **Loŋoolu/ londoolu** | | | | | |
|  | Ali nene ye kirikiroo kibaaroo moyile baŋ? | 1. haa 2. hani | | | | |
|  | Ali ye a moyi miŋtoo walla muŋ feŋ ne la (niŋ j1=haa ti)? | 1. rajoo  2. tele to  3. karanbuŋo to  4. fondinkee kuroo to  5. jaaralilaa la waraŋ dulaa doo to  6. waraŋ dulaa doolu to, i safee | | | | |
|  | Kirikiroo ka soto ñaadii le (suŋolu)?(niŋ j1=haa ti) | 1. I niŋ saasaatoo ye i laa 2. Suusuulaa kindiroo 3. Tunbu kindiroo \| 4. Domo lolliriŋo domoo 5. Faŋ seneyaabaliyaa 6. Tulu domoo 7. Ka i jaa tiloo koto 8. Waraŋ doo koteŋ 9. Waraŋ muŋ maŋ loŋ | | | | |
|  | Kirikiroo ka suutee ñaadii le? (nij Q1=haa ti) | 1. Kuŋ dimiŋo 2. Kono dimiŋo 3. Jarajaroo (nenoo) 4. Bala bataa 5. Daa seeyaa 6. Kono dimiŋo 7. foonoo 8. waraŋ doo koleŋ, i safee 9. waraŋ muŋ maŋ loŋ | | | | |
|  | Kirikiroo se ñaatoosii ñaadii le? (faŋ tankandi a ma ñaadii) (niŋ j1=haa ti) | 1. sankee 2. bori junjuntaa (ex. yotox) 3. bori ŋorobontinmaa 4. lankaraŋo booroo 5. ñaama baroo 6. dondika yiriwaa duŋ 7. jii loodulaa sukki 8. foñoo sumaa joraŋo 9. waraŋ doo 10. waraŋ muŋ maŋ loŋ | | | | |
| 1. **maañaalu** | | | | | | |
|  |  | Wo sahayaata | Sonata a ma | Maŋ feŋ loŋ jee | Maŋ soŋ wo la | Wo maŋ sahayaa |
|  | Kirikiroo se moo bee muta noo le |  |  |  |  |  |
|  | Kirikiroo ka moo faa le |  |  |  |  |  |
|  | Kirikiroo se jaara noo le hani I maŋ a bori taa |  |  |  |  |  |
|  | Kirikiroo se ñaatoosii noo le |  |  |  |  |  |
|  | Kirikiroo ñanta loŋ na foloo janniŋ I be a jaarala |  |  |  |  |  |
|  | Kirikiroo jaaraaloo ñanta banna le fereŋ |  |  |  |  |  |
| 1. **keeñaa muŋul si soto katamaroo kono (jaarali ñiniŋ)** | | | | | | |
|  | Fo a ye ali muta le niŋ waatiŋ kono? | 1. haa 2. hani | | | | |
|  | Ali ye a loŋ ñaadii le? (sawuŋ niŋ j1=hani ti) | 1, juubeeroo to jaarali buŋo to  2, booroolu minnu sotota  3, keeñaalu  4, waraŋ doo koteŋ, i safee | | | | |
|  | Ali nene taata jaaraaloola le? (sawuŋ niŋ j1=hani ti) | 1. haa 2. hani | | | | |
|  | Munne ye a tinna ali maŋ taa jaaraaloo la? (sauter si Q3=Oui) | 1. Bulu dassoo (kodoo) 2. Naa solo (waaloo) 3. janfoo (dulaa) 4. Amaŋ wossi 5. Maŋ taa dulaa soto 6. waraŋ doo koteŋ, i safee | | | | |
|  | I taata muŋ too le ke I jaara? (niŋ j3=haa ti) | 1. jaara li buŋo to 2. jaara li buŋ baa to 3. daalaa jaara li dula 4. Moo fiŋ jaaralilaa yaa 5. Fansuŋ jaaraloo 6. Boori wafii dulaa 7. waraŋ doo koteŋ, i safee | | | | |
|  | Ali ye waati jelu le sii kabiriŋ mantaalu foloota a ye la janniŋ ali be taala jaarali ñininna ? (nij Q3=haa ti ) | 1. wo luŋo faŋo 2. tili kiliŋ 3. wo tili fuloo kono 4. waraŋ doo koteŋ: _________________________ 5. waraŋ muŋ maŋ loŋ | | | | |
|  | Ali niŋ jaarali buŋo muŋ ka dookuo kef o moo se waali boo noo jee taa to (waati jaalu la se kee noo suuwoolu niŋ jaarali buŋo ñiŋ teemoolu ? | 1. Miŋ dasata wati kiliŋ la (< 60 min) 2. Miŋ wasata wati kiliŋ la (> 60 min) | | | | |
