# Supplementary material for: Malaria prevention knowledge, attitudes, and practices (KAP) among adolescents living in an area of persistent transmission in Senegal: Results from a cross-sectional study
Source: PLoS One. 2022 Dec 1;17(12):e0274656. doi: 10.1371/journal.pone.0274656 (PMC9714833; doi:10.1371/journal.pone.0274656)
Supplement: S1 Table — Tables are ordered as follows: Table A Logistic regression of the determinants related to malaria knowledge among adolescents; Table B Logistic regression of the factors associated with attitude of the participants towards malaria; Table C Logistic regression of the factors associated with malaria prevention practice; Table D Logistic regression of factors associated with malaria care-seeking behavior. (DOCX) [file pone.0274656.s005.docx]

Table A Logistic regression of the determinants related to malaria knowledge among the study participants

|  |  | **Knowledge level** | |  |  |  |
| --- | --- | --- | --- | --- | --- | --- |
| **Variable** | **Category** | **Low** | **High** | **COR (95% CI)** | **AOR (95% CI)** | **P** |
| **Characteristics of the participants** |  | n (%) | n (%) |  |  |  |
| **Age (in years)** | 10—14 | 125 (65.1%) | 67 (34.9%) | 1 | 1 |  |
|  | 15 – 19 | 83 (66.4%) | 42 (33.6%) | 0.94 (0.59-1.52) | 0.90 (0.51-1.59) | 0.705 |
| **Sex** | Female | 109 (59.2%) | 75 (40.8%) | 1 | 1 |  |
|  | Male | 99 (74.4%) | 34 (25.6%) | 0.50 (0.31-0.81) | 0.40 (0.24-0.68) | **0.001***** |
| **Education level** | None | 35 (87.8%) | 05 (12.2%) | 1 | 1 |  |
|  | Koranic | 32 (76.2%) | 10 (23.8%) | 2.25 (0.70-7.29) | 2.67 (0.79 -9.08) | 0.116 |
|  | Primary | 125 (61.3%) | 79 (38.8%) | 4.55 (1.71-12.11) | 5.43 (1.91-15.46) | **0.002***** |
|  | Secondary | 15 (50.0%) | 15 (50.0%) | 7.2 (2.2 - 23.42) | 10.41 (2.98-36.40) | **0.000***** |
| **Wealth index** | Highest | 45 (64.3%) | 25 (35.7%) | 1 | 1 |  |
|  | Fourth | 42 (60.9%) | 27 (39.1%) | 1.16 (0.58 -2.30) | 1.29 (0.61- 2.70) | 0.502 |
|  | Middle | 45 (67.2%) | 22 (35.5%) | 0.88 (0.43-1.78) | 0.77 (0.36-1.65) | 0.507 |
|  | Second | 40 (64.5%) | 22 (35.5%) | 0.99 (0.48-2.02) | 1.02 (0.46-2.28) | 0.953 |
|  | Lowest | 36 (73.5%) | 13 (26.3%) | 0.65 (0.29-1.45) | 0.87 (0.36-2.07) | 0.751 |
| **Characteristics of the households** |  | | | | | |
| **Education level** | None | 80 (58.4%) | 57 (41.6%) | 1 |  | 1 |
|  | Koranic | 80 (72.1%) | 31 (27.9%) | 0.54 (0.32-0.93) | 0.58 (0.32-1.05) | 0.073 |
|  | Primary | 43 (70.5%) | 18 (29.5%) | 0.59 (0.31-1.12) | 0.56 (0.28-1.14) | 0.109 |
|  | Secondary | 03 (50.0%) | 03 (50.0%) | 1.40 (0.27-7.22) | 0.89 (0.21-3.70) | 0.872 |

*** Significant at the 0.01 level

Table B Logistic regression of the factors associated with attitude of the study participants towards malaria

|  |  | **Malaria attitude level** | | | |  |
| --- | --- | --- | --- | --- | --- | --- |
| **Variable** | **Category** | **Positive** | **Negative** | **COR (95% CI)** | **AOR (95% CI)** | **P** |
| **Characteristics of the participants** |  | **n(%)** | **n(%)** |  |  |  |
| **Age (in years)** | 10 -14 | 114 (59.4%) | 78(40.6%) | 1 | 1 |  |
|  | 15 - 19 | 73(58.4%) | 52 (41.6%) | 0.96(0.61 - 1.52) | 0.72(0.41-1.24) | 0.235 |
| **Sex** | Female | 107 (58.2%) | 77 (41.8%) | 1 | 1 |  |
|  | Male | 80 (60.2%) | 53 (39.8%) | 1.09(0.69 - 1.71) | 0.95(0.58 - 1.57) | 0.850 |
| **Education level** | No education | 28 (68.3%) | 13(31.7%) | 0.43(0.13 - 1.38) | 0.47(0.13 - 1.79) | 0.272 |
|  | Koranic | 17 (40.5%) | 25(59.5%) | 0.13(0.04 - 0.43) | 0.14(0.36 - 0.55) | **0.005***** |
|  | Primary | 117 (57.4%) | 87 (42.6%) | 0.27(0.10 - 0.73) | 0.24(0.70 - 0.79) | **0.019**** |
|  | Secondary | 25 (83.3%) | 05 (16.7%) | 1 | 1 |  |
| **Wealth index** | Highest | 57 (81.4%) | 13 (18.6%) | 4.21(1.85 - 9.59) | 3.49(1.48 - 8.28) | **0.004***** |
|  | Fourth | 35(50.7%) | 34(49.3%) | 0.99(0.47 - 2.06) | 0.97(0.45 - 2.11) | 0.941 |
|  | Middle | 43(64.2%) | 24(35.8) | 1.72(0.81 - 3.65) | 1.53(0.68 - 3.44) | 0.304 |
|  | Second | 27(43.6%) | 35(56.4%) | 0.74(0.35 - 1.57) | 0.71(0.31 - 1.59) | 0.401 |
|  | Lowest | 25(51.0%) | 24(49.0%) | 1 | 1 |  |
| **Characteristics of the household head** |  | | | | | |
|  | No education | 75(54.7%) | 62(45.3%) | 1 | 1 |  |
|  | Koranic | 66(59.5%) | 45(40.5%) | 1.21(0.73 - 2.01) | 1.28(0.73 - 2.25) | 0.388 |
|  | Primary | 43(70.5%) | 18(29.5%) | 1.97(1.04 - 3.77) | 1.84(0.90 - 3.78) | 0.097 |
|  | Secondary | 3(50.0%) | 3(50.0%) | 0.83(0.16 -4.25) | 0.47(0.08 - 2.80) | 0.409 |

** Significant at the 0.05 level

*** Significant at the 0.01 level

Table C Logistic regression of the factors associated with malaria prevention practice of the study participants

|  |  | **Malaria prevention practice** | | | |  |
| --- | --- | --- | --- | --- | --- | --- |
| **Variable** | **Category** | **Good** | **Poor** | **COR (95% CI)** | **AOR (95% CI)** | **P** |
| **Characteristics of the participants** |  | **n(%)** | **n(%)** |  |  |  |
| **Age (in years)** | 10-14 | 74 (31.9%) | 158 (68.1%) | 1 | 1 |  |
|  | 15-19 | 46 (28.9%) | 113 (71.1%) | 0.87 (0.56 - 1.35) | 0.69 (0.38 - 1.25) | 0.225 |
| **Sex** | Female | 66 (30.1%) | 153 (69.9%) | 1 | 1 |  |
|  | Male | 54 (31.4%) | 118 (68.6%) | 1.06 (0.69 -1.63) | 1.24 (0.72 - 2.14) | 0.434 |
|  | No education | 20 (48.8%) | 21 (51.2%) | 1 | 1 |  |
|  | Koranic school | 16 (38.1%) | 26 (61.1%) | 0.65 (0.27 -1.55) | 0.64 (0.24 -1.70) | 0.372 |
| **Educational level** | Primary school | 59 (28.9%) | 145 (71.1%) | 0.43 (0.22 - 0.87) | 0.40 (0.18 - 0.91) | **0.028**** |
|  | Secondary school | 09 (30.0%) | 21 (70.0%) | 0.45 (0.17 -1.22) | 0.36 (0.11 - 1.13) | 0.079 |
| **Wealth index** | Highest | 38 (48.7%) | 40 (51.3%) | 1 | 1 |  |
|  | Fourth | 25 (32.0%) | 33 (67.9%) | 0.50 (0.26-0.95) | 0.42 (0.19 - 0.89) | **0.024**** |
|  | Middle | 15 (20.0%) | 64 (81.0%) | 0.25 (0.12-0.51) | 0.34 (0.16 - 0.72) | **0.005**** |
|  | Second | 21 (26.9%) | 57 (73.1%) | 0.39 (0.20 - 0.76) | 0.42 (0.19 - 0.90) | **0.027**** |
|  | Lowest | 21 (26.9%) | 57 (73.1%) | 0.39 (0.20 - 0.76) | 0.55 (0.23 - 1.32) | 0.179 |
| **Attitude level** | Negative | 31 (23.9%) | 99 (76.1%) | 1 | 1 |  |
|  | Positive | 73 (39.0%) | 114 (61.0%) | 2.04 (1.25 - 3.37) | 1.89 (1.08 - 3.30) | **0.026**** |
| **Knowledge level** | Low | 74 (35.6%) | 134 (64.4%) | 1 | 1 |  |
|  | High | 30 (27.5%) | 79 (72.5%) | 0.69 (0.41 - 1.14) | 0.80 (0.46 - 1.39) | 0.435 |
| **Characteristics of the household head** |  | | | | | |
| **Educational level** | No education | 40 (24.4%) | 124 (75.6%) | 1 | 1 |  |
|  | Koranic school | 58 (43.0%) | 77 (57.0%) | 2.33 (1.43 - 3.83) | 1.85 (1.04 - 3.28) | **0.036**** |
|  | Primary school | 17 (21.2%) | 63 (78.7%) | 0.84 (0.44 - 1.59) | 0.88 (0.44 - 1.75) | 0.707 |
|  | Secondary school | 05  (55.6%) | 04 (44.4%) | 3.88 (0.99-15.16) | 7.02(0.73 - 67.51) | 0.092 |

** Significant at the 0.05 level

Table D Logistic regression of the factors associated with malaria care-seeking behavior of the study participants

|  |  | **Malaria care seeking practices** | | | |  |
| --- | --- | --- | --- | --- | --- | --- |
| **Variable** | **Category** | **Good**  **n (%)** | **Bad**  **(n%)** | **COR (95% CI)** | **AOR (95% CI)** | **P** |
| **Age (in years)** | 10--14 | 135(77.1%) | 40(22.9%) | 1 | 1 |  |
|  | 15 - 19 | 74(68.5%) | 34(31.5%) | 0.64(0.38 - 1.11) | 0.57(0.32 - 1.02) | 0.056 |
| **Sex** | Female | 123(58.8%) | 38(51.3%) | 1 | 1 |  |
|  | Male | 86(41.1%) | 36(48.6%) | 0.74(0.43 - 1.26) | 0.68(0.38 - 1.19) | 0.174 |
| **Wealth index** | Highest | 50(82.0%) | 11(18.0%) | 1 | 1 |  |
|  | Fourth | 51(78.5%) | 14(21.5%) | 0.80(0.33 - 1.94) | 0.87(0.36 - 2.14) | 0.756 |
|  | Middle | 40(70.2%) | 17(29.8%) | 0.52(0.22 - 1.23) | 0.52(0.22 - 1.26) | 0.147 |
|  | Second | 35(60.3%) | 23(39.7%) | 0.33(0.14 - 0.78) | 0.35(0.15 - 0.80) | **0.013**** |
|  | Lowest | 33(78.6%) | 9(12.2%) | 0.81(0.30 - 2.16) | 0.86(0.32 - 2.32) | 0.772 |

** Significant at the 0.05 level
